# Feasibility of embryonic-natural orifice transluminal endoscopic surgery for submucosal tumors with mucosal preservation in beagle model

**DOI:** 10.3389/fsurg.2026.1772776

**Published:** 2026-04-09

**Authors:** Han Lin, Chuan-Shen Jiang, Dong-Gui Hong, Long-Ping Chen, Yan Zhuang, Wen Lin, Zan-Xuan Qiu, Xin-Yi Zheng, Meng-Yao Xu, Li-Li Wu, Yang-Xin Xie, Wen Wang, Xiao-Jian He, Da-Zhou Li

**Affiliations:** 1 Fuzong Clinical Medical College of Fujian Medical University, Fuzhou, China; 2 Department of Gastroenterology, 900th Hospital of PLA Joint Logistic Support Force, Fuzhou, China

**Keywords:** beagle model, embryonic-natural orifice transluminal endoscopic surgery, gastric submucosal tumors (SMTs), gastrointestinal submucosal tumors, mucosal preservation technique

## Abstract

**Background and aims:**

While endoscopic resection techniques such as endoscopic submucosal excavation (ESE) or endoscopic full-thickness resection (EFTR) enable the removal of gastrointestinal muscularis propria–originating submucosal tumors (MP-SMTs), these procedures still present certain limitations, including risks of perforation and hemorrhage. Therefore, we developed a novel embryonic-natural orifice transluminal endoscopic surgery (E-NOTES) approach designed to achieve complete resection of MP-SMTs while preserving mucosal integrity. This study evaluated its feasibility in beagle model.

**Methods:**

Twelve healthy beagles underwent E-NOTES using a gastric endoscope. Simulated lesions were created on the serosal surface of the gastric anterior wall. The muscularis propria and serosal layers were resected while maintaining mucosal integrity. Simulated lesion size, operative time, intraoperative hemorrhage, intra-/ postoperative complications were recorded and analyzed. Postoperative endoscopy at 60 days and histopathological examination were performed to assess outcomes.

**Results:**

All procedures were successfully completed without conversion to laparoscopy. The mean simulated lesion diameter was 28.1 ± 7.7 mm, and mean operative time was 64.3 ± 5.5 min. Minor intraoperative bleeding (< 5 mL) occurred in 7/12 (58.3%) dogs and was controlled endoscopically. No severe complications (perforation, peritonitis, or major bleeding) occurred. All animals resumed oral intake within 24 h. Endoscopy on postoperative day 60 confirmed complete mucosal healing, and histopathology verified the complete excision of the targeted full-thickness muscularis propria and the preservation of mucosal layers.

**Conclusion:**

In this preclinical canine model, E-NOTES enabled safe and complete resection of gastric MP-SMTs with intact mucosa in a beagle model, demonstrating minimal invasiveness, low complication rates, and rapid recovery. These findings support further technical refinement and cautious evaluation in subsequent translational studies.

## 1 Introduction

With the widespread application of endoscopic examinations and the development of endoscopic ultrasonography (EUS), the detection rate of gastrointestinal submucosal tumors (SMTs) has increased significantly in recent years (1). Most SMTs are asymptomatic and incidentally detected during imaging or endoscopic examinations. However, depending on their size and location (e.g., at the esophagogastric junction), they can cause severe complications, including direct cardia compression and secondary megaesophagus. Furthermore, conventional endoscopy or radiological modalities often fail to confirm their pathological characteristics. Except for gastrointestinal stromal tumors (GISTs, which have malignant potential), the majority of gastric SMTs are benign (2). Due to the difficulty in obtaining a definitive preoperative diagnosis, resection is frequently performed as a combined diagnostic and therapeutic approach. Approximately 23.1% of SMTs exhibit an extraluminal growth pattern, while the remainder primarily grow intraluminally (3).

For intraluminal SMTs, endoscopic resection techniques such as endoscopic submucosal dissection (ESD) and ESE are well-established, enabling *en bloc* resection of mucosa- or submucosa-originating tumors under minimally invasive conditions (4, 5). In contrast, SMTs arising from the muscularis propria (MP) layer or with extraluminal growth are unsuitable for ESD: their deep location and tight adherence to the MP layer increase the risks of perforation and hemorrhage, reduce the complete resection rate, and impose high technical demands (6). Thus, alternative approaches—including EFTR, laparoscopic-endoscopic cooperative surgery (LECS), or laparoscopic wedge resection—are typically required (7–9).

According to the American Society for Gastrointestinal Endoscopy (ASGE) guidelines, EFTR can achieve complete resection of SMTs originating from the deep gastric wall (10–12). However, previous studies report that major complications of EFTR (e.g., delayed perforation, intra-abdominal infection) occur in up to ∼20% of cases in some series, and these adverse events significantly prolong hospital stays (13, 14). LECS and laparoscopic wedge resection provide a wider surgical field but often remove excessive normal gastric tissue, potentially resulting in structural deformation and complications such as postoperative bleeding, gastroesophageal reflux, or delayed anastomotic stenosis (15). Furthermore, similar to the effects observed in partial gastrectomy, this excessive resection and alteration of gastric anatomy may compromise gastric accommodation and predispose patients to long-term functional disturbances such as postprandial fullness and impaired nutrient absorption (16).

In recent years, E-NOTES as an innovative technique utilizing the umbilicus as the access route, has gained increasing attention (17–21). Unlike EFTR (which performs full-thickness resection from the mucosal side to the serosal side) and LECS (which requires dual access via both endoscopy and laparoscopy), E-NOTES uses a single umbilical entry to directly expose lesions from the peritoneal cavity, allowing the endoscope to perform precise, layer-by-layer dissection of the gastric wall. For MP-SMTs, this retrograde resection approach (from the serosa inward toward the muscular layer) achieves complete tumor removal while preserving mucosal integrity, thereby minimizing infection and complication risks (22). Additionally, E-NOTES offers advantages including a broad operative field, minimal peritoneal trauma, simplified defect management, and an aesthetically concealed umbilical incision.

To address the limitations of current surgical approaches for MP-SMTs, this study evaluated the feasibility and safety of E-NOTES for the resection of gastric MP-SMTs in a beagle model, with a focus on verifying mucosal preservation, resection completeness, and perioperative safety.

## 2 Materials and methods

### 2.1 Experimental animals

Twelve healthy beagles (aged 8–12 months, mean body weight 15.1 ± 1.47 kg) were purchased from Fuzhou Zhenhe Laboratory Animal Technology Co., Ltd. All animals underwent a 7-day quarantine period to confirm absence of infectious diseases (e.g., canine parvovirus, distemper) and meet institutional health criteria. Preoperatively, animals were fasted for 24 h and deprived of water for 12 h to reduce gastric content-related intraoperative risks. All experimental procedures were conducted in compliance with the Declaration of the Helsinki Convention on the Use and Care of Animals and approved by the Institutional Review Board of the 900TH Hospital of Joint Logistics Support Force (approval No. 2023-53).

### 2.2 Instruments and medications

The instruments used in this study included gastric endoscopy (GIF-H260: distal end outer diameter 9.8 mm, working length 1,030 mm, instrument channel diameter 2.8 mm; or GIF-Q260: distal end outer diameter 9.2 mm, working length 1,030 mm, instrument channel diameter 2.8 mm; Olympus Optical Co., Ltd.), transparent cap (Olympus Medical Systems), insulation-tipped knife (KD-650L; Olympus Medical Systems), Hook knife (KD-620QR; Olympus Medical Systems), GoldKnife (MK-T-1-235; Micro-Tech Endoscopy USA),polypectomy snares (STIFFBx/10; Boston Scientific Corporation), hot biopsy forceps (KD-410LR; Olympus Medical Systems), hemostatic clamp (Boston Scientific Corporation), VIO 300D device (ERBE Elektromedizin GmbH, Germany), and 12-mm disposable trocars (Hangzhou Kangji Medical Equipment Co., Ltd.). Medications included Zoletil® 50 (Virbac, France), normal saline, and indigo carmine dye for submucosal injection and tissue staining. All endoscopic instruments were thoroughly cleaned and disinfected by the central sterilization unit and subsequently sterilized using ethylene oxide before each procedure.

### 2.3 Anesthesia and intraoperative monitoring

General anesthesia was induced by intramuscular injection of Zoletil® 50 (Tiletamine 125 mg + Zolazepam 125 mg; Virbac, Carros, France) at a dose of 5–10 mg/kg. Anesthesia depth was monitored by the absence of corneal reflex and tail pinch response; additional doses (2 mg/kg) were administered if voluntary movement was observed. After the animals were placed in the supine position, vital signs including heart rate and respiration were continuously monitored throughout the procedure.

### 2.4 Surgical procedure

**Figure 1:**
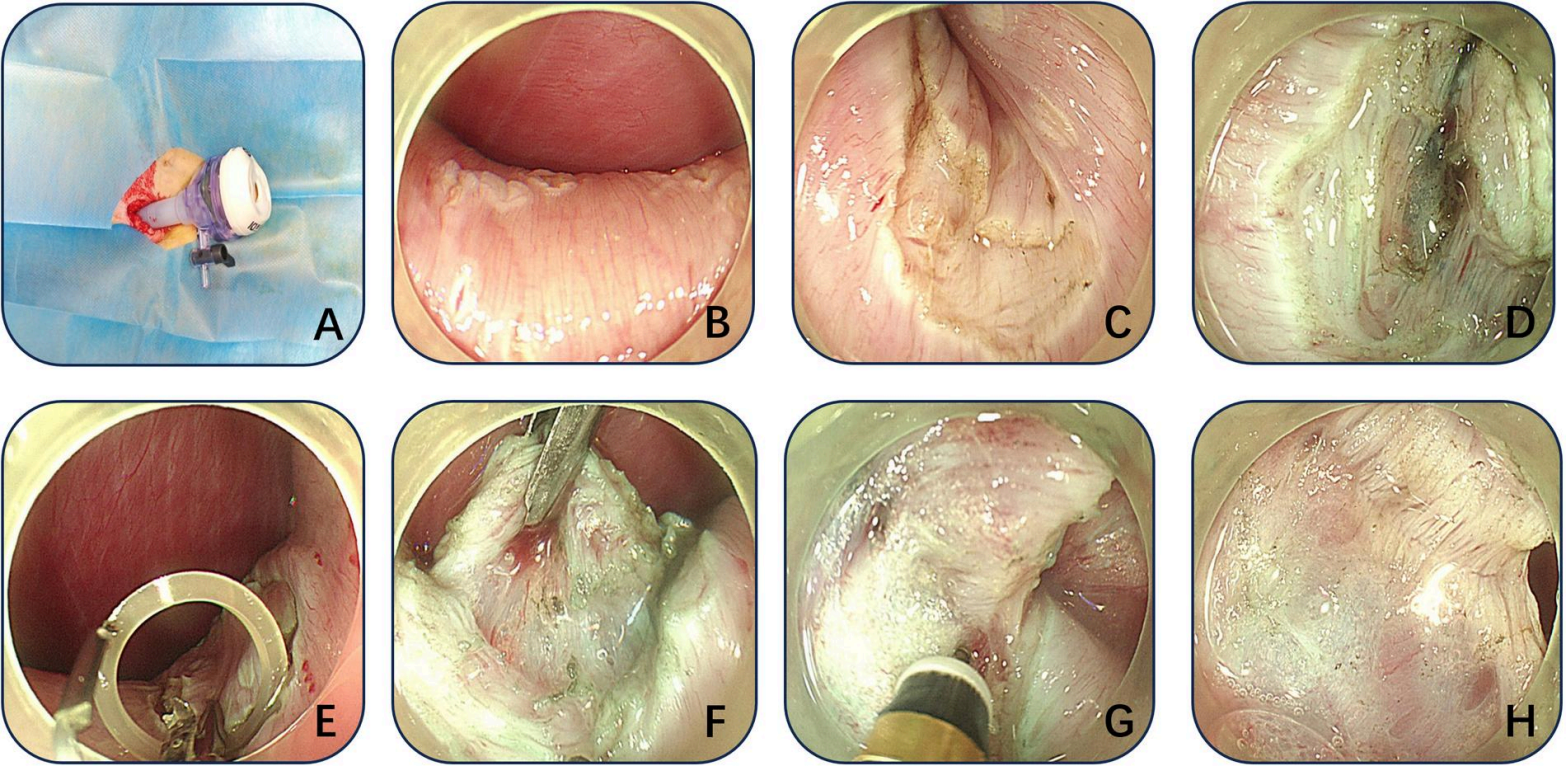
Schematic of transumbilical endoscopic muscularis resection **(A–H)**. **(A)** Insertion of the trocar through the transumbilical incision to establish an endoscopic channel; **(B)** Circular marking of the lesion boundary; **(C)** Incision of the serosa and muscularis propria; **(D)** Submucosal injection of saline containing indigo carmine to create a submucosal cushion; **(E,F)** Continuous exposure of the surgical field using clip-and-rubber band traction; **(G,H)** Dissection of the muscularis propria with the Hook knife, with repeated submucosal injections to maintain separation.

**Figure 2:**
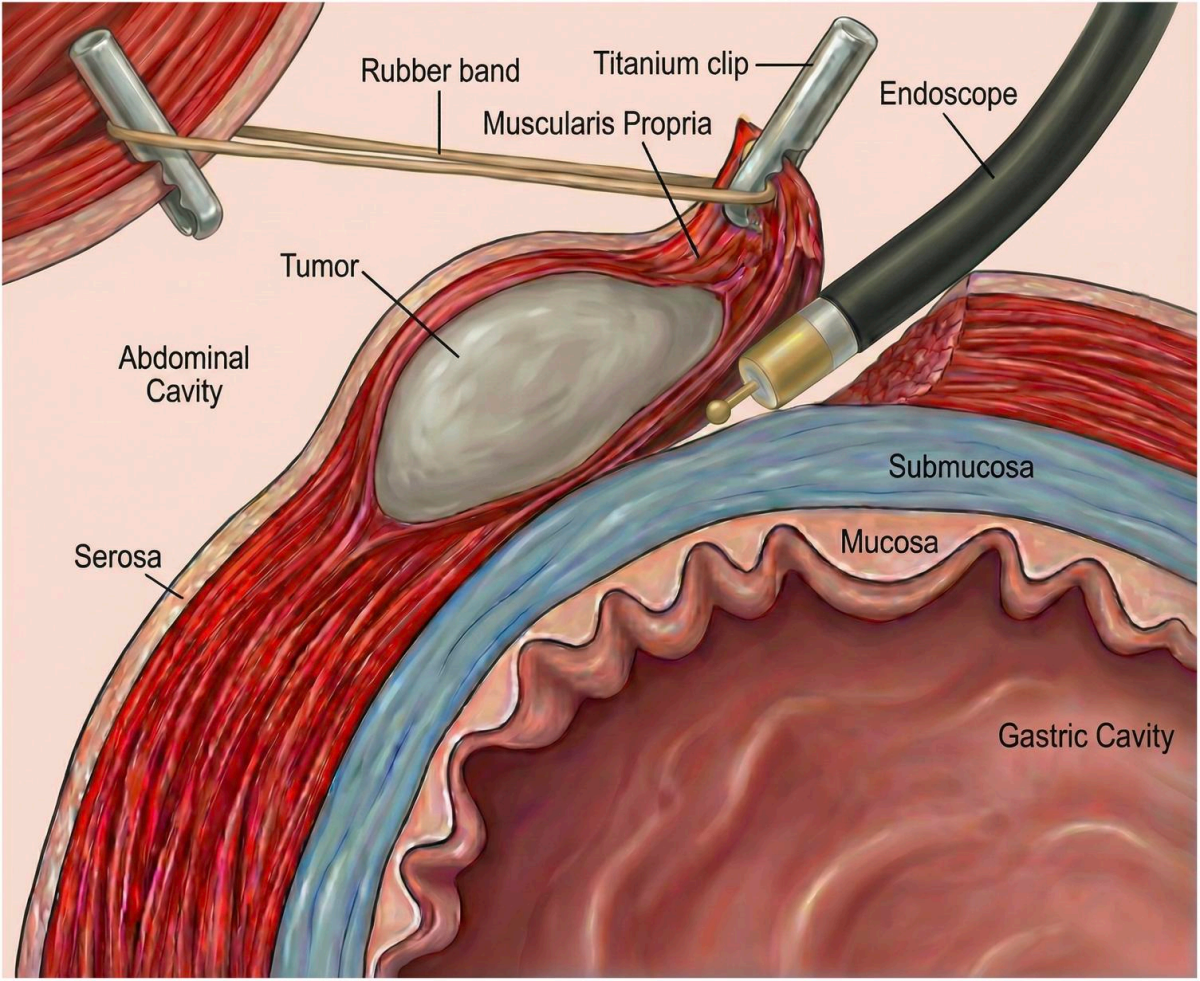
Schematic illustration of the E-NOTES mucosal-preserving resection technique.

**Figure 3:**
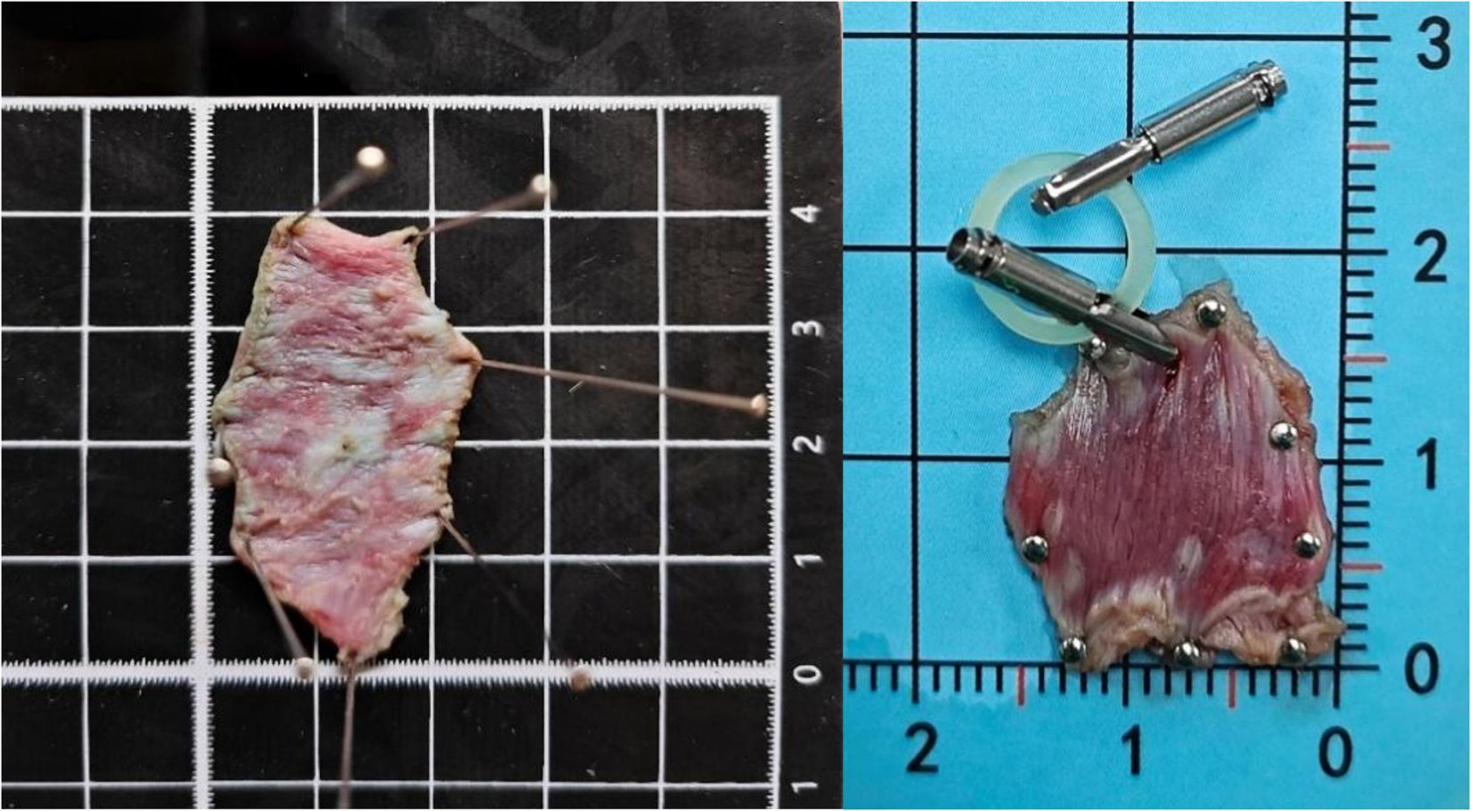
Resected serosal and muscularis propria specimens after the procedure.

Under sterile conditions, a 1.0 cm arc-shaped skin incision was made at the umbilicus, through which a 12-mm disposable trocar was inserted to establish the endoscopic channel. After CO₂ insufflation to create pneumoperitoneum, the gastroscope was introduced via the trocar (Figure 1A). Simulated lesions (15–40 mm) were marked on the serosal side of the gastric anterior wall using the Dual knife at 5–10 mm intervals to delineate the resection boundary (Figure 1B). An incision was then made in the serosa and muscularis propria (Figure 1C), while preserving the mucosa and submucosa. Indigo carmine–stained saline was injected into the submucosa to facilitate separation (Figure 1D). A clip-and-rubber band traction technique was applied to expose the surgical field (Figures 1E,F). The target muscularis tissue was dissected with the Hook knife under continuous traction, with repeated submucosal injections to maintain tissue separation (Figures 1G,H). Additional representative images are shown in Figure 2. After complete resection, the muscular layer, clips, and rubber band were retrieved through the trocar (Figure 3). The abdominal cavity was inspected for bleeding or injury to surrounding organs, the trocar was withdrawn, and the umbilical incision was sutured to complete the procedure. Finally, endoscopic evaluation of the gastric cavity was performed to check for bleeding or perforation.

### 2.5 Postoperative care and observation

Postoperatively, animals were housed in a quiet environment. Animals were allowed to resume oral intake once they fully recovered from anesthesia. To objectively evaluate the natural recovery process and the unassisted resumption of feeding, no routine postoperative analgesic medications were administered. Daily assessments included general condition, feeding, activity, defecation, and wound healing (infection, umbilical hernia, etc.). Endoscopy was performed on postoperative day 60 to evaluate healing (ulceration, fistula, stricture).

### 2.6 Data recording and analysis

Primary outcomes included the technical success rate, defined as the proportion of procedures completed without conversion to laparoscopy; the complete targeted tissue resection rate, confirmed by histopathological examination showing the intactness of the full-thickness muscularis propria and serosal layers within the resected specimen; and the mucosal preservation rate, verified via endoscopy to confirm intact mucosa postoperatively. Secondary outcomes encompassed the operative time (measured from trocar insertion to skin closure), intraoperative bleeding volume (estimated using the suction canister and gauze weight), postoperative complication rate (including complications such as perforation, bleeding, infection, and hernia), time to resume oral intake, and laboratory parameters [specifically the white blood cell [WBC] count and C-reactive protein [CRP] level]. Peripheral venous blood was collected preoperatively and on days 1, 3, and 7 postoperatively to measure blood counts and CRP.

### 2.7 Statistical analysis

Data were analyzed using SPSS 26.0 software (IBM Corp., Chicago, IL, USA). Continuous variables were presented as mean ± standard deviation (SD) or median [interquartile range (IQR)] based on normality testing (Shapiro–Wilk test). Categorical variables were presented as counts (percentages). Since this was a descriptive animal study, no inferential statistical tests (e.g., t-test, chi-square test) were performed; focus was placed on summarizing procedural and outcome data. *P* < 0.05 was defined as statistically significant if comparisons were conducted.

## 3 Results

### 3.1 Intraoperative outcomes

**Figure 4:**
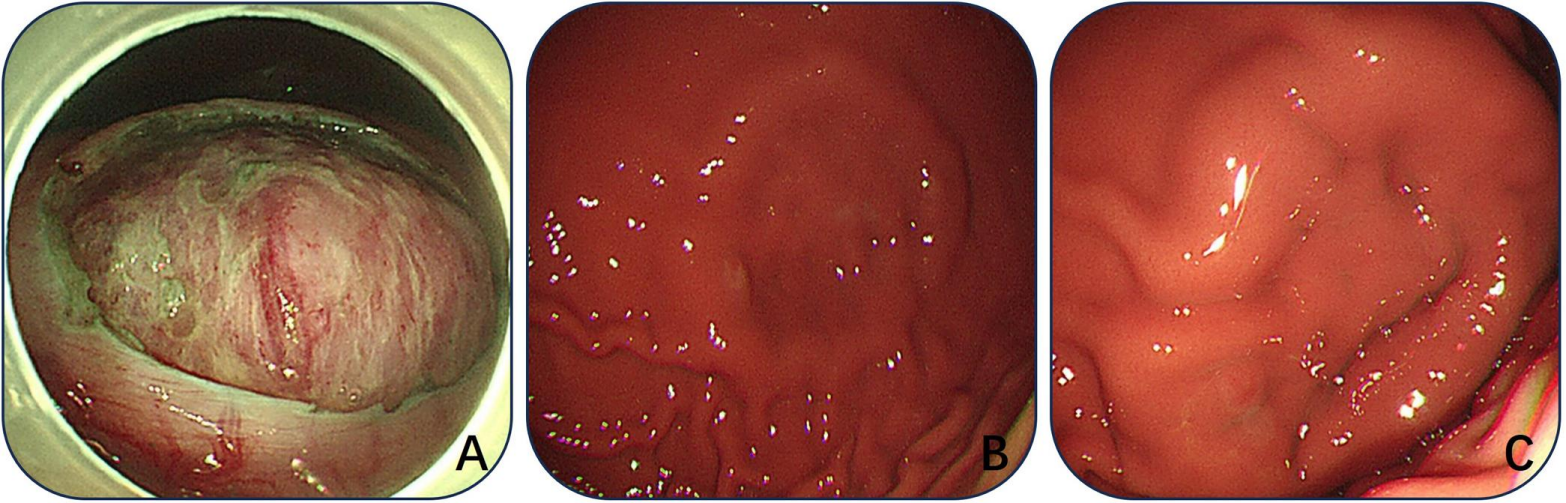
Postoperative evaluation of the abdominal and gastric cavity. **(A)** Postoperative intra-abdominal inspection; **(B)** Postoperative intragastric inspection; **(C)** Intragastric inspection at 60 days postoperatively.

All 12 beagles successfully underwent E-NOTES, simulated MP-SMT resection, and umbilical incision closure, with no conversion to laparoscopy (technical success rate, 100%). No vascular, neural, or visceral injuries were observed during trocar placement. Intraoperative bleeding was observed in 7 animals (58.3%), all classified as minor (<5 mL) and effectively controlled using hot biopsy forceps without additional hemostatic interventions. The mean maximum diameter of resected specimens was 28.1 ± 7.7 mm (range, 14–40 mm), with 2 specimens (16.7%) < 20 mm, 7 (58.3%) 20–30 mm, and 3 (25.0%) > 30 mm. The mean total operative time was 64.3 ± 5.5 min, consisting of 56.5 ± 4.6 min for lesion excision and 7.8 ± 1.7 min for incision creation and closure. Histopathological examination of all specimens confirmed the presence of intact serosal and full-thickness muscularis propria layers, bordered by a small amount of submucosal tissue, demonstrating the *en bloc* excision of the targeted muscular wall (complete targeted resection rate, 100%), and immediate postoperative gastroscopy verified intact mucosa and submucosa at the resection site (mucosal preservation rate, 100%; Figures 4A,B). Detailed intraoperative and postoperative outcomes are summarized in Table 1.

**Table 1:** Surgical outcomes and complications in beagle dogs (*n* = 12).

Parameter	Value
Simulated lesion size (mm), *n* (%)	
<20	2 (16.7%)
20–30	7 (58.3%)
>30	3 (25.0%)
Maximum specimen diameter (mm, mean ± SD)	28.1 ± 7.7
Surgical outcomes, *n* (%)	
Successful resection	12 (100%)
En bloc resection	12 (100%)
Operative time (min, mean ± SD)	
Total operative time	64.3 ± 5.5
Resection time	56.5 ± 4.6
Incision and closure time	7.8 ± 1.7
Intraoperative events, *n* (%)	
Minor bleeding (<5 mL, managed endoscopically)	7 (58.3%)
Major bleeding	0 (0%)
Perforation	0 (0%)
Peritonitis	0 (0%)
Adjacent organ injury	0 (0%)
Postoperative outcomes, *n* (%)	
Resumed oral intake within 24 h	12 (100%)
Transient reduced intake/activity (<48 h)	2 (16.7%)
Wound healing without complications	12 (100%)
Wound infection/hernia	0 (0%)
Delayed bleeding/perforation	0 (0%)
Peritonitis/abscess	0 (0%)
Endoscopic healing at day 60	12 (100%)
Laboratory findings, *n* (%)	
Elevated WBC/CRP on days 1–3	8 (66.7%)
Elevated WBC/CRP on day 7	0 (0%)

Data are presented as mean ± SD where applicable.

### 3.2 Postoperative recovery and complications

All animals resumed oral intake within 24 h postoperatively. Two beagles (16.7%) exhibited transient reductions in food consumption (≈50% of baseline) and activity levels within the first 24 h, which resolved spontaneously without intervention by 48 h postoperatively. No severe postoperative complications (e.g., perforation, delayed bleeding, peritonitis, or abscess) were observed. All umbilical incisions achieved primary healing within 7 days, with no signs of infection (redness, exudate) or umbilical hernia. Fecal occult blood tests remained negative in all animals throughout the 60-day follow-up period, with no melena or hematochezia observed.

### 3.3 Laboratory findings and long-term follow-Up

**Figure 5:**
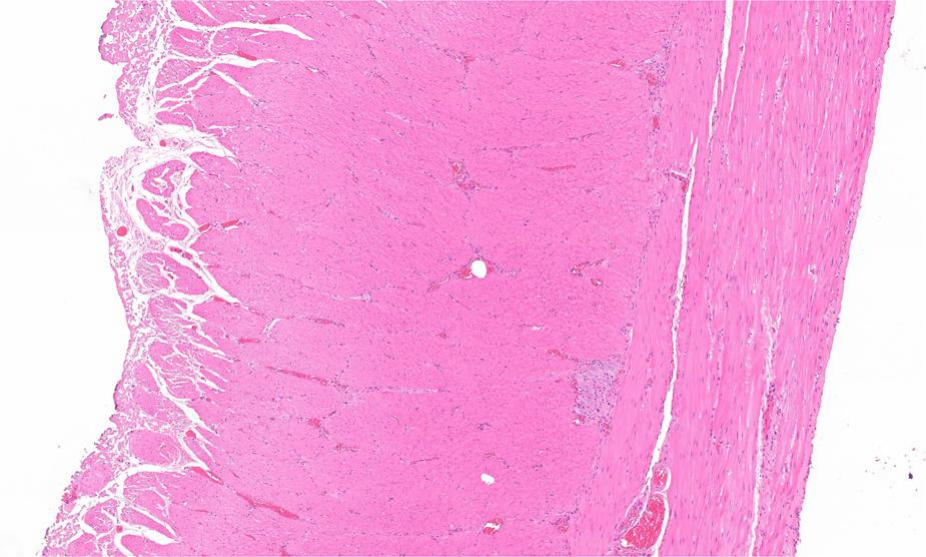
Resected specimen showing intact muscularis propria and serosa.

Peripheral venous blood tests showed that 8 animals (66.7%) had transient elevations in WBC count and CRP level within 72 h postoperatively, consistent with mild surgical stress or inflammatory responses. All WBC and CRP values returned to within the normal reference ranges (WBC: 6.0–17.0 × 10^9^/L; CRP: <10 mg/L) by postoperative days 3–7 without anti-inflammatory or antibiotic treatment. Endoscopic evaluation on postoperative day 60 revealed complete mucosal healing at the resection site, with no ulceration, fistula formation, or structural abnormalities of the gastric wall (Figure 4C). Histopathological re-examination of the gastric wall at the resection site confirmed intact mucosal and submucosal layers, with no residual muscularis propria tissue (Figure 5).

## 4 Discussion

This study demonstrates that E-NOTES enables safe and effective resection of gastric MP-SMTs in a beagle model, with 100% technical success, complete targeted resection, and mucosal preservation rates. While situated as an early technical exploration within a controlled preclinical setting, these findings address critical limitations in current surgical paradigms (23) and underscore the unique advantages of E-NOTES for future clinical translation.

The most notable advantage of E-NOTES is its ability to preserve mucosal integrity, a key gap in existing endoscopic and surgical approaches. EFTR recommended by the American Society for Gastrointestinal Endoscopy (ASGE) for deep gastric SMTs (10–12), requires full-thickness gastric wall excision, which disrupts the mucosal barrier. This disruption contributes to major complications such as delayed perforation and intra-abdominal infection, with incidences reaching ∼20% in some series (13, 14), and often necessitates delayed postoperative feeding (>48 h) (24, 25). LECS and laparoscopic wedge resection provide a wider surgical field but often remove excessive normal gastric tissue, potentially resulting in structural deformation and complications such as postoperative bleeding, gastroesophageal reflux, or delayed anastomotic stenosis (15). Furthermore, similar to the effects observed in partial gastrectomy, this excessive resection and alteration of gastric anatomy may compromise gastric accommodation and predispose patients to long-term functional disturbances such as postprandial fullness and impaired nutrient absorption (16). In contrast, E-NOTES' retrograde resection (from serosa to muscular layer) avoids mucosal injury entirely, thereby serving as a potential mucosa-preserving alternative that prioritizes anatomical preservation: our results showed no mucosal defects at immediate or 60-day postoperative endoscopy, and all animals resumed oral intake within 24 h, the absence of gastric leakage or peritoneal contamination in this animal model suggests that mucosal preservation may mitigate some theoretical risks associated with intragastric or transluminal procedures.

E-NOTES also addresses practical challenges of other minimally invasive techniques. LECS requires dual access and coordination between endoscopic and laparoscopic teams (8), increasing procedural complexity and operative time. E-NOTES, by contrast, uses a single transumbilical port for endoscopic access, simplifying the workflow and reducing the mean total operative time to 64.3 ± 5.5 min—comparable to or shorter than reported EFTR and LECS times (26, 27). Additionally, the umbilical incision achieves nearly scarless healing. Although direct evidence regarding patient aesthetic expectations specifically following gastric SMT resection is limited, studies on other transumbilical procedures, such as single-incision laparoscopic cholecystectomy, show that 40%–50% of younger patients cite postoperative scar appearance as a key factor in surgical decision-making (28). This cross-disciplinary evidence strongly suggests that the aesthetically concealed incision makes E-NOTES a compelling option for patients with cosmetic expectations alongside clinical safety needs.

Our technical modifications further enhance E-NOTES’ reproducibility. We used indigo carmine-stained saline injection to create a submucosal cushion—facilitating precise separation of the muscularis propria from the submucosa—and applied clip-and-rubber band traction to maintain a clear surgical field. These adjustments, rarely described in prior E-NOTES studies (19, 21), likely contributed to the low intraoperative bleeding rate (58.3% of cases with <5 mL bleeding, controlled via hot biopsy forceps) and 100% complete targeted resection rate, supporting the technique's feasibility with standardized training.

Despite these strengths, this study has three key limitations. First, the beagle model has anatomical differences from humans: canine gastric wall thickness (0.2–0.3 cm) is thinner than that of humans (0.3–0.5 cm) (29), which may underestimate intraoperative bleeding risks and dissection difficulty when applied to human patients. Future studies using porcine models—with gastric wall thickness closer to humans—or *ex vivo* human tissue specimens are needed to better simulate clinical conditions. Second, we only tested simulated MP-SMTs (14–40 mm) located on the gastric anterior wall. In clinical practice, MP-SMTs may arise on the posterior wall (adjacent to the pancreas or spleen) or have dense adhesions to surrounding organs (7), which could increase operative complexity and complication risk. Third, the learning curve of E-NOTES—which integrates advanced endoscopic and laparoscopic skills—was not evaluated. Previous studies on endoscopic techniques have shown that proficiency directly impacts complication rates (23); thus, establishing structured training protocols (e.g., simulator practice, supervised animal experiments, and case-by-case mentoring) will be critical for safe clinical adoption.

## 5 Conclusion

In summary, this study demonstrates the feasibility and safety of E-NOTES for the resection of gastric MP-SMTs, highlighting its advantages of minimal invasiveness, rapid recovery, high mucosal preservation rates, and favorable cosmetic outcomes. Nevertheless, limitations related to animal models, tumor complexity, and the technical learning curve remain. Therefore, the current findings should be regarded as hypothesis-generating rather than practice-changing. Further studies involving larger animal models, procedural standardization, and carefully designed early-phase clinical evaluations are required before any clinical application can be considered. If these developmental milestones are achieved, E-NOTES offers a potential path toward enhancing the surgical management of gastric MP-SMTs.

## Data Availability

The raw data supporting the conclusions of this article will be made available by the authors, without undue reservation.
